# Problems of modern approaches to management of early pregnancy failure

**DOI:** 10.4274/tjod.79059

**Published:** 2015-12-15

**Authors:** Müberra Namlı Kalem, Ziya Kalem, Ebru Yüce, Ayla Eser, Zehra Candan İltemir Duvan

**Affiliations:** 1 Turgut Özal University Faculty of Medicine, Department of Obstetrics and Gynecology, Ankara, Turkey; 2 Gürgan Clinic, In Vitro Fertilization Center, Ankara, Turkey

**Keywords:** Early pregnancy failure, ultrasonography, human chorionic gonadotropin hormone, Methotrexate

## Abstract

In the last 20 to 30 years, early diagnosis of pregnancy has markedly decreased ectopic pregnancy-related maternal mortality, and the necessity for surgical treatment. With modern approaches in the treatment of ectopic pregnancy, surgical therapy has been replaced by medical therapy and medical treatment by spontaneous follow-up in appropriate cases. However, this current trend has led to some problems, including the maximization of ultrasonographic interpretations, misunderstandings in serial human koryonik gonadotropin hormon measurements, and complications due to inappropriate methotrexate use. The aim of the present study was to review the literature relating to the diagnosis and follow-up of early pregnancies, to underline some of the important considerations, and to help avoid possible iatrogenic errors.

## INTRODUCTION

In the last 20 to 30 years, early diagnosis of pregnancy has markedly decreased ectopic pregnancy-related maternal mortality, and the necessity for surgical treatment. With modern approaches in the treatment of ectopic pregnancy, surgical therapy has been replaced by medical therapy and medical treatment by spontaneous follow-up in appropriate cases. However, this current trend has led to some problems, including the maximization of ultrasonographic interpretations, misunderstandings in serial beta human koryonik gonadotropin hormon (β-hCG) measurements, and complications due to inappropriate methotrexate use^(1)^.

In early symptomatic ectopic pregnancies, which present with pain and bleeding, the primary aim is the maintenance of viability, and determination of location within the shortest time; the goal is to avoid late diagnosis of ectopic pregnancy, and to cause no harm to an intrauterine pregnancy. Emergency approaches in the maintenance of viability and determination of location in early pregnancies lead to unnecessary interventions or the possibility of false diagnoses. On the other hand, delays lead to serious problems, as does failure to diagnose an existing ectopic pregnancy^(2)^.

Various strategies have been established for monitoring the early period of pregnancies for clarifying location and maintaining viability using transvaginal ultrasonography (USG) and hCG measurements; however, an optimal strategy does not exist to predict the course of the pregnancy. Therefore, unnecessarily frequent follow-ups or interventions are performed during this period, even in normal pregnancies. The approach of the physician and the avoidance of superfluous interventions play an important role in decreasing parents’ anxiety^(3)^.

The aim of the present study was to review the literature relating to the diagnosis and follow-up of early pregnancies, to underline some of the important considerations, and to help avoid possible iatrogenic errors.

## DIAGNOSTIC METHODS USED FOR EARLY PREGNANCIES

The main visualization method used in early pregnancy is transvaginal USG,^(4)^ in which false positive and false negative rates are high^(5)^. USG does not have diagnostic value in early pregnancy when hCG values are below 1500 IU/mL^(6)^. At higher values, the experience of the physician and resolution of the device gain importance.

Besides USG, hCG measurement is the most important parameter that assists diagnosis in early pregnancy. A single hCG measurement has no diagnostic value in determining the location and viability of a pregnancy; serial hCG measurements are needed. A single hCG measurement can only be helpful when the hCG threshold value (discriminatory zone) is precisely determined. The hCG threshold value indicates that a single viable intrauterine pregnancy does not exist when an intrauterine sac cannot be identified using transvaginal USG. The discriminatory zone is currently accepted to be 1500 IU/mL^(1,7)^.

Progesterone can be used to determine the viability of an intrauterine pregnancy. In a patient with pain or bleeding, a single progesterone measurement can confirm non-viability when USG diagnosis is not sufficient. The results of a meta-analysis that included 26 studies indicated that a pregnancy could be accepted as 99% not viable when progesterone is determined at an interval of <3.2 ng/mL to 6 ng/mL. Ectopic pregnancy, early loss or normal early pregnancy cannot be differentiated using progesterone levels. Consequently, it should not be used for this purpose^(8)^.

Initiation of early pregnancy follow-up increases incorrect considerations. In early pregnancies, both USG and hCG have high error margins. An hCG value below 500 mIU/mL can be considered as a sign of an abnormal pregnancy^(9)^. Chung et al.^(10)^ ascertained that an intrauterine pregnancy existed in more than 50% of cases when hCG values were above the threshold and USG did not reveal an intrauterine sac. These results were supported by later studies^(11)^.

Attempts to resolve the difficulties of determining location and viability in early pregnancies are defined as pregnancy of unknown location (PUL) and pregnancy of uncertain viability (PUV).

PUL is considered when a sac cannot be visualized in the uterus or adnexa, although hCG indicates a value above the threshold. PUL does not define a diagnosis or pathology, but a process. The Royal College of Obstetricians and Gynaecologists (RCOG) (2006) defined PUV pregnancy as gestational sac <20 mm and invisible embryo or yolk sac, or embryonic CRL <6 mm and absence of fetal heart activity^(12)^.

A study by Bottomley et al.,^(13)^ which was conducted with 1442 pregnancies, demonstrated that transvaginal USG findings before the 35^th^ day from the beginning of the last menstrual period (LMP) could define a pregnancy as PUL, between the 35^th^ and 41^st^ days they could define PUV, and after the 42^nd^ day they could confirm a viable intrauterine pregnancy. The possibility for precision in determining viability increases progressively till the 49^th^ day, and plateaus after the 49^th^ day. USG performed after the 49^th^ gestational day with consideration to the LMP reduces false diagnoses without increasing morbidity that might result from the late diagnosis of ectopic pregnancy.

Ultrasonographic diagnoses of ectopic pregnancies have been replaced by the determination of an adnexal mass, rather than the absence of an intrauterine sac. Casikar et al.^(14)^ reported USG findings of ectopic pregnancy as follows: a blob sign (60%), a bagel sign (20%), and a gestational sac, fetal pole or fetal heart beat visualization (13%). Transvaginal USG is accepted as the gold standard in ectopic pregnancy diagnoses, with a 74% sensitivity and 99.9% specificity^(15)^.

Gestational age is the best determinant in the evaluation of early pregnancies. Bottomley et al.^(13)^, recommended that USG should not be used before the 49^th^ day in asymptomatic pregnancies to avoid inessential follow-ups and analyses. In a study conducted with assisted reproductive technology (ART) pregnancies, it was reported that a gestational sac had to be seen using USG by the time a pregnancy of known gestational age reached five weeks five days, and USG should not be performed before 5.5 weeks of gestation in asymptomatic ART pregnancies^(1,16)^.

## INCREASING HCG VALUES

The first studies related to serial hCG measurements were conducted with asymptomatic infertile patients; it has long been understood that hCG has to increase two fold every two days^(17)^. The first serial hCG study in spontaneous pregnancies was performed in 20 symptomatic patients, and it was reported that the least hCG increase needed for viability determination had to be 66%^(18)^.

Barnhart et al.^(19)^ determined this value as 53% in their 2004 study. The study included 287 patients, and the confidence interval was 99%, i.e., if an increase in the hCG value is less than 53%, the possibility of a viable pregnancy is 1%.

The results of subsequent studies have varied greatly. In a study that included 200 cases of ectopic pregnancy, the initial hCG elevations in 60% of the patients were determined to be more than 53%, which has been defined as the lower limit for a viable intrauterine pregnanc^(20)^. During follow-ups of 340 symptomatic patients in 2011, ectopic pregnancy developed in ten (16%) of the 63 women who had normal β-hCG elevations^(21)^.

Variations in hCG levels are more frequent in in vitro fertilization (IVF) pregnancies than in spontaneous pregnancies. Pregnancies with higher initial hCG levels tend to continue, and fetal loss rates are higher in those with lower initial hCG values^(22)^. When embryos were transferred on the 3^rd^ day, hCG values determined on the 13^th^ and 15^th^ days were found to be higher compared with 5^th^ day blastocyst transfers; however, proportions of hCG elevations were observed to be similar^(23)^. In another study, hCG above 150 on the 15th day, and a ratio of the 22^nd^ day hCG to the 15^th^ day hCG above 15 indicated a normal pregnancy course, with a specificity of 94% and a sensitivity of 47%^(24)^. Proportions in hCG elevations between the 14^th^ and 16^th^ days after oocyte pick-up (OPU) in 6021 IVF pregnancies were demonstrated to correlate with the rate of live births^(25)^.

The time needed for hCG levels to increase two fold was reported not to differ between spontaneous-conception pregnancies and IVF gestations;^(26)^ the same authors reported soon afterwards that the time for hCG levels to increase two fold did not differ between 48 IVF single pregnancies, and 50 IVF multiple pregnancies^(26)^. In a study that investigated 224 IVF single-, 135 IVF twin-, and 32 IVF triplet pregnancies, total hCG levels in multiple pregnancies were found to be higher compared with the single-pregnancy group; however, the proportions of the elevations were similar in all groups^(27)^. In this respect, there are insufficient data regarding heterotopic pregnancies.

## HUMAN CHORIONIC GONADOTROPIN HORMONE VALUES THAT DECREASE OR PLATEAU

hCG values that decrease or plateau have been shown to indicate a non-viable pregnancy^(28)^. The exceptions are laboratory errors and Ovarian hyperstimulation syndrome (OHSS). Hemodilution in OHSS resulting from the shift of extravascular fluid to the intravascular system may lead to an unusual lowering of hCG levels^(29)^.

After the determination of a non-viable pregnancy, hCG may also serve as a guide when considering intervention or follow-up. Intervention is required in early intrauterine losses and in ectopic pregnancies that do not regress spontaneously, whereas spontaneously regressing ectopic pregnancies and early intrauterine losses can be followed up with no intervention in association with hCG measurements^(30)^.

A linear proportion does not exist in hCG decreases. Decreases in hCG levels were followed up until they fell below 5 m IU/mL in 710 patients with spontaneous abortion (2004), and the hCG decrease was shown to be ‘quadratic.’ Decreases are more rapid in those with high initial hCG values; the rate of decrease slows as the initial hCG values decrease^(31)^. In induced abortions, hCG decreases more rapidly compared with spontaneous abortions^(32)^. Table 1 summarizes the lowest decreases in hCG levels for clinical use in spontaneously resolving pregnancies that did not require intervention^(30)^.

The decline of hCG has no diagnostic value per se, but it may be used during follow-ups. The following general rules have to be kept in mind while interpreting hCG declines^(18,19,20,21)^:

- Follow-up is essential until hCG level decreases below 5 m IU/mL.

- If the decrease in hCG is not sufficient, intervention (cavity aspiration) is needed to discriminate between ectopic pregnancy and non-viable intrauterine pregnancy.

- A regressing ectopic pregnancy may rupture despite lowering of hCG levels.

- Clinical follow-up has to be continued in cases of PUL, where there is a suspicion of an ectopic pregnancy, despite the resolution of hCG.

- Clinical signs should always be primarily considered, rather than laboratory results, and an intervention should not be planned based on hCG investigations alone.

## FOLLOW-UP IN PREGNANCY OF UNKNOWN LOCATION

The term pregnancy of unknown location (PUL) should be used when a gestational sac cannot be visualized in the uterus or adnexa, despite hCG values being above the threshold. PUL is not a diagnosis or pathology, but a stage. According to the classification of Barnhart et al.,^(33)^ a pregnancy defined as PUL may exist with the following consequences: intrauterine pregnancy, ectopic pregnancy, persistent PUL, and failed PUL. Patients who progress to ectopic pregnancy carry a high risk, and those who progress to an intrauterine pregnancy or terminate spontaneously are considered to have low-risk PUL^(34)^.

Eight percent to 14% of patients with PUL progress to an ectopic pregnancy. This group is the most important with regard to maternal mortality and morbidity, and early diagnosis thus gains great importance during follow-up^(35,36)^.

Progesterone measurements can also be used in PUL to estimate the course of follow-up. A serum progesterone level below 20 nmol/L indicates a non-viable pregnancy,^(35)^ a progesterone level above 25 nmol/L supports viability, and levels above 60 nmol/L indicate the strong possibility of a viable pregnancy^(12)^. It was demonstrated in a meta-analysis that progesterone measurements may support non-viability, but would not help to discriminate between an ectopic pregnancy or failed PUL^(37,38)^.

Proportions of hCG elevations are more precious than progesterone levels in terms of pregnancy^(39)^. Levels of hCG in a series analysis are expected to increase by 53% to 66% every 48 hours in a normal intrauterine pregnancy; an increase in a proportion above 66% can confirm a normal pregnancy at 95%^(40)^. A 48-hour hCG ratio above 1.66 indicates the maintenance of an intrauterine pregnancy, and weekly ultrasonographic checks are appropriate for determining location. Cut-off values that show a pregnancy will not continue range between 0.79 and 0.89. In this situation, pregnancy is immediately followed-up and weekly hCG measurements are performed; pregnancy is expected to terminate within four to six weeks without any complications^(41)^. Values between 0.79 to 1.66 define a suboptimal increase of hCG and generally have to be interpreted as ectopic pregnancies^(42)^.

A 48th hour hCG value determined lower than 87% of the initial level in PUL determines the spontaneous resolution of a pregnancy with a 90% sensitivity and specificity; these patients should be followed up^(41)^.

A sufficient rate of hCG decline is not diagnostic for a non-viable intrauterine pregnancy; some ectopic pregnancies may also exist with similar declining patterns. In the follow-ups of 200 ectopic pregnancies, hCG levels reduced similarly to those seen in spontaneous abortion in 8% of patients^(43)^. Six (3%) of 214 pregnant women who had appropriate decreases during serial measurements were reported to have had ectopic pregnancies^(21)^.

Clinical signs are also considered in the follow-up of PUL. The existence of bleeding in early pregnancy, and specifically its continuation for more than three days, increases the possibility of ectopic pregnancy^(44)^. Signs of pelvic pain and bleeding during early pregnancies should prompt physicians to consider ectopic pregnancy; however, these signs are not specific and may also exist in the loss of early intrauterine pregnancies. Moreover, ruptured ectopic pregnancies may be completely asymptomatic. Therefore, laboratory findings, USG results, and clinical signs should be interpreted together, in order to avoid late diagnosis. In the study of Condous et al.,^(36)^ the time to diagnosis of ectopic pregnancy was reported to range from two to 25 days after first considering it as PUL; the median time was found as five days.

The best approach for patients with pregnancies of unknown location who are stable and whose pregnancies tend to continue, is simply to follow-up. In this way, the probability of causing harm to a possible intrauterine pregnancy will be avoided^(10,35)^.

In cases of PUL, the hCG discriminatory zone is not solely sufficient for determining viability and location^(45)^. In determining viability, the most conservative threshold values have to be selected for hCG elevation proportions, and hCG-progesterone single measurements^(46)^. Condous et al.^(47)^ evaluated 1003 patients with PUL, both retrospectively and prospectively, in a broad-spectrum study. In their analysis, pregnancies that should have been terminated, according to the ASRM recommendations of that time, were followed up without being terminated. During these follow-ups, no problems occurred as result of the delayed diagnosis of ectopic pregnancy. The authors concluded that uterine curettage had to be performed for diagnostic considerations in PUL to avoid the risk of iatrogenic termination of pregnancy that have potential for viability.

If a pregnancy of unknown location is absolutely determined to be non-viable, it is pertinent to wait for the spontaneous regression of the pregnancy, without making any intervention^(33)^. When pregnancy terminates in this way, it is impossible to make an exact distinction between intrauterine and extrauterine pregnancies. If this discrimination is to be made, endometrial curettage material has to be investigated histopathologically. The presence of chorionic villi indicates intrauterine pregnancy, and their absence indicates ectopic pregnancy. Methotrexate administration is essential in the absence of chorionic villi in endometrial curettage material and in cases of rising hCG levels, even after the procedure.

However, based on suppositions, methotrexate administration has progressively increased in patients who have not been precisely diagnosed. Methotrexate use is accepted to be inappropriate in intrauterine or extrauterine pregnancies that would already be spontaneously resorbed, or in cases where the possibility of an intrauterine viable pregnancy has not been precisely excluded; this approach may cause the following risks^(1,48,49)^:

- Intrauterine loss or abortion.

- Maintenance of pregnancy and teratogenic adverse effects.

- Exposure of the mother to systemic adverse effects.

- Negative effects of methotrexate on ovarian reserve.

- Medicolegal problems.

Another point that needs to be considered is the inaccurate diagnosis of ectopic pregnancies, and the existence of inappropriate long-term methotrexate use data in the diagnostic and therapeutic statistical records. In the future, these faulty data would lead to higher incidences of ectopic pregnancies being reported and a greater success for methotrexate to inaccurately endure in the treatment.

Persistence may also exist in cases of non-viable PUL due to the persistence of trophoblastic tissue viability or its spontaneous regression. Asymptomatic high levels of hCG have to be followed-up urgently; however, particularly in cases of prolonged bleeding or risk of infection, the decision for surgery or medical curettage can be made based on the patient’s signs^(41,42,45)^.

## FOLLOW-UP OF PREGNANCY OF UNCERTAIN VIABILITY

In the PUV definition of the RCOG 2006, a gestational sac (GS) size of 20 mm was required for the visualization of embryo, and a crown-rump length (CRL) of 6 mm was required for the visualization of heartbeats^(12)^. Various studies were performed in the following years to determine cut-off values, and similar results were obtained.

The criteria of the American College of Radiologists (ACR) from the 2009 report can be summarized as follows^(50)^: The embryo is initially observed as a linear exogenous increase in density between the yolk sac and gestational sac. It is possible to be visualized when the GS is 8 mm in size, but it is essential for it to be observed when the GS is 16 mm. If cardiac activity is absent when the embryo is >5 mm, a missed abortion diagnosis can be made. In normal gestation, the sac grows 1 mm each day. These values were suggested to be considerably challenging for obstetricians because they are radiology data that were derived from high-resolution ultrasound devices.

In the study of Pexsters et al.,^(51)^ intra- and inter-observer variations were determined in 20% of the evaluations of CRL and GS, even in measurements performed by experienced physicians. It is important to know about this wide range of variations because it indicates that a CRL value measured as 20 mm could be simultaneously measured between 16 to 24 mm.

In a review that investigated the reliability of USG data in the diagnosis of early pregnancy losses, an empty sac with a GS size of ≥25 mm, and the absence of yolk sac with a GS of ≥20 mm, were determined to be criteria with a specificity of 95%, and a reliability of 95%. However, high-quality prospective studies are not available in this respect^(52)^.

Abdallah et al.^(53)^ reported two multi-center studies (2011) related to the determination of viability using USG. The results of these studies, which respectively included 1060 and 359 patients, can be summarized as follows: corresponding values were determined with regard to growth rates of gestational sacs in the groups of patients with viable and non-viable PUV; a daily or weekly cut-off value related with viability or non-viability could not be determined; rates of embryonic growth showed differences; a CRL growth rate of below 0.2 mm/day indicated non-viability with a specificity of 100%; a CRL growth rate of at least 1.4 mm/week was in accordance with viability; GS value may not change for a several days in a viable pregnancy, but no difference was determined in embryonic tissues between two follow-up tests, which indicated non-viability; a slow-growing embryo also indicated that it would be lost^(54)^.

In the 2012 National Institute for Clinical Excellence (NICE) guidelines, the criteria for early pregnancy loss were determined as follows: all cases have to be examined under transvaginal (TV)-USG; pregnancy is accepted to be non-viable in TV-USG findings if a yolk sac or fetal pole is invisible despite a GS of ≥25 mm, or if fetal cardiac activity is absent despite a CRL of ≥7 mm; if there is a suspicion related with the diagnosis and/or if the patient demands repeated tests, the patient has to be reevaluated at least at one-week intervals without any preparation for a medical or surgical curettage; if no growth is detected in GS or CRL in this repeated test, or if the embryonic tissues cannot be visualized, the pregnancy has to be accepted as non-viable.

An empty gestational sac with a size of <25 mm in the TV-USG or a CRL <7 mm in the absence of fetal cardiac activity must be accepted as doubtful signs, and the patient has to be monitored for at least a seven-day period. At the second follow-up, if the gestational sac is empty or if the embryo is invisible, despite the visualization of yolk sac in the first check, the pregnancy has to be accepted non-viable. The third check has to be performed one week after, if needed^(55)^.

In the wide-spectrum miscarriage treatment trial (MIST) study of Trinder et al.,^(56)^ spontaneous follow-up, medical treatment, and surgical treatment in the management of early pregnancy losses were compared. The results of this study indicated high success rates in all three approaches, and the complication rates were determined to be low.

Doubilet et al.^(57)^ reported USG criteria for non-viability in early pregnancies: the pregnancy has to be accepted as non-viable if the fetal heart beat is absent when CRL is ≥7 mm, and if the embryo is invisible when GS is ≥25 mm; an embryo existing with heart beat must be visible two weeks after the visualization of a sac, which does not include a yolk sac, or 11 days after the visualization of a sac that includes a yolk sac; the pregnancy has to be accepted as non-viable if these visualizations cannot be made.

In Bourne’s^(58)^ interpretation of the 2015 NICE guidelines, non-diagnostic doubtful signs related to pregnancy loss were summarized as follows:

- CRL <7 mm and heartbeats are invisible.

- GS between 16 to 24 mm and invisible embryo.

- An embryo with absent heartbeat 7 to 13 days after the visualization of a sac that does not include yolk sac.

- An embryo with invisible heartbeat 7 to 10 days after the visualization of a sac that includes yolk sac.

- An invisible embryo six weeks after the last menstrual period.

- An embryo is invisible despite the amnion being visualized with the yolk sac.

- Yolk sac >7 mm.

- Small sac size compared with the embryo (difference of mean sac diameter (MSD)-CRL <5 mm).

## CONCLUSION

The time needed for the determination of viability and location in early pregnancies, whether or not they are symptomatic, causes anxiety for families and physicians. This period has been reduced by the improvement of technology, the greater USG resolutions, and the widespread use of hCG measurements. However, this has led to unnecessary interventions and also to possible harm of intrauterine pregnancies. Various studies have been conducted in recent years in this respect, and the standardization of clinical approaches is being made, but exact criteria for the prediction of the course of pregnancies have not yet been determined. The aims of these approaches are to avoid the excluding an ectopic pregnancy and not to cause harm to normal intrauterine pregnancies. Well-planned further studies are needed to determine an optimal strategy.

## Figures and Tables

**Table 1:**
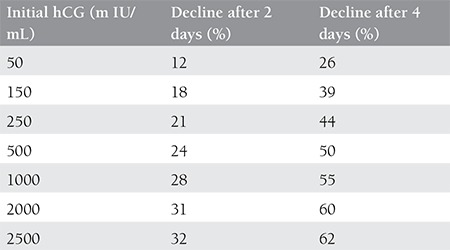
Table 1
